# Integrating mental health: the last frontier?

**DOI:** 10.5334/ijic.1058

**Published:** 2012-09-28

**Authors:** Rebecca Chong, Weng-Mooi Tan, Loong-Mun Wong, Jason Cheah

**Affiliations:** Community Mental Health, Agency for Integrated Care, Republic of Singapore; Community Mental Health, Agency for Integrated Care, Republic of Singapore; Agency for Integrated Care, Republic of Singapore; Agency for Integrated Care, Republic of Singapore

The World Health Organization (WHO) has estimated that globally, more than 150 million people today suffer from depression. The WHO further estimates that mental, neurological, and substance use disorders account for 14% of the global burden of disease. In Singapore, major depressive disorders (MDD) is the most common psychiatric disorder. One in 16 people in Singapore have suffered from MDD at some point in their lifetime [1]. While the knowledge base for integrating care for persons afflicted with chronic diseases and the frail elderly increases exponentially, it seems less obvious in the literature on integrating care for persons suffering from chronic mental illnesses. There have been numerous published reports and papers on the increasing global burden of mental illnesses, prompting the World Health Organization (WHO) to pronounce that psychiatric diseases will become the main cause of morbidity in the developed world in the not-too-distant future. A number of developed nations have already established excellent mental health programmes which address issues concerning access to appropriate and timely acute medical treatment, to crisis management, and long-term (residential and home-based) care. While this is a step in the right direction, there is still a gap in our understanding of how best to integrate mental health programmes and services with the “mainstream” physical healthcare delivery system. It is also a well-known fact that anywhere between 20% and 40% of patients suffering from chronic medical diseases (e.g. diabetes mellitus, hypertension, chronic renal failure, etc.) have also concommitent depression (clinical or sub-clinical). There is also increasing evidence that dementia is becoming a more prevalent cause of significant morbidity, especially amongst the frail elderly. All these point to the pressing need to integrate mental health services. The big question is how best to do so in a cost-effective and efficient manner. Lastly, the important role of the care-giver should not be forgotten and the need for adequate and on-going support and respite for these persons.

The journey to integrate care for persons suffering from mental illnesses is fraught with many challenges. One is the **uncertainty and misalignment** of the vision and goals of the healthcare delivery system. It is usually difficult to imagine (or “visualise”) how the entire system would appear as providers across the care continuum have partial perspectives from their “silo” positions. There are also **organisational barriers** to integrated mental healthcare. These barriers are often exacerbated by the “divide” between health and social care services, the absence of a robust shared electronic clients’ record, and the persisting weakness of commissioning. For care to be undertaken by both health professionals and social agencies, there is a need to (i) create an integrated network of services integrating social and health elements, and (ii) develop a system to share information, through standardised assessment tools and protocols. Another challenge is **the shift in focus from clinical care to capability building**. It is always a challenge in the acquisition of new skill-sets especially across the different sectors within the care continuum. Lastly, the **transformation of responsibilities** from a traditional paternalistic care approach to a self-advocating care can pose major roadblocks. Historically, providers have taken all the important decisions in the best interest of their patients, but with the new model of care, providers need to have a paradigm shift whereby they work in partnership with people with mental illnesses. This is a fundamental re-orientation for health and social care professionals.

The integration of care for mental health conditions is highly complex because the patients’ needs require a spectrum of social and healthcare services which may change over time as the psychiatric condition progresses. In Singapore, the journey to integrate care for persons suffering from mental disorders started in 2007 when the first National Mental Health Blueprint was established. Key gaps, such as mental health services driven primarily by acute hospitals and tend to be hospital-centric; inadequate General Practitioners (GP) willing to treat mental health; insufficient coordinated efforts between key stakeholders (social and health); and the looming presence of stigma. Hence, there was an urgency to build a network of care and support systems to enable integrated community living, maximising individual potential, and promoting aging in place. In 2012, to take the National Mental Health Blueprint to the next step, Singapore’s Ministry of Health adopted a population health approach—The Community Mental Health Strategy—to address the health needs of mental health patients (including those at risk) and developing the services and support. It takes into consideration the entire range of factors that determine health which includes employment and income, social support, education and housing. Based on this approach, the identified priority target population are those diagnosed with depression and/or with anxiety, dementia, schizophrenia, as well as those at risk including the elderly who are living alone.

For successful implementation of an integrated mental healthcare delivery network, three strategic themes have been articulated to guide the development over the next 5 years: 1) Capability Building; 2) Capacity Building; and 3) Communication and Engagement. These initiatives will be implemented in a phased approach. For a start, **6 key initiatives** have been identified to be the main focus** within the next 2 years.** The first is the capacity and capability gap. There is a current lack in capacity, capability and range of mental health services in the community. It is mainly run by charities and non-government organizations. The Agency for Integrated Care (AIC), which is a national coordinating body, has been tasked to build capability and capacity, seed new services and build a multi-layered and sectorial service to maintain patients safely in the community whilst giving them the best possibility to lead active and productive lives.

Some of the key initiatives are:

- **Community Resource and Engagement Support Teams (CREST)** reach out to residents to provide a) education and information on common types of Mental Health and illness; b) encourage clients to seek early help; and c) where needed, provide a referral processes linking to services based on clients’ and caregivers’ needs; and d) escalate cases to other Community support mechanisms. The community remains the best source of information and resource to extend help for such cases with their close proximity and established relationship with the communities. The religious VWO and social groups are already providing first-line pastoral and social counseling for troubled individuals.
- **Community Intervention Teams (COMIT),** consist of a team of allied health professionals, provide a) education, counseling and support for family members; b) follow-up care and support by “para-counsellors”; and c) coordinate and integrate care with medical professionals. COMIT supports and complements the GPs, providing intervention and support, so as to keep clients well and productive.
- **Assessment Shared Care Teams (ASCAT) are physician-led.** The main objective is to stabilize patients through specific interventions and to provide treatment for patients who maybe have severe conditions but can still be safely managed in the outpatient setting.
- **Another key initiative is to build up Manpower Capability.** This necessitates investments in developing expertise (training), skills and knowledge on mental health issues. Scholarships to encourage more to take up Community Mental Health as a professional will create a pipeline of professionals in the short- and medium-term.

The second is an information and communication gap on mental health conditions and services. Currently, the general public and patients are often unaware of how, when and where to seek help or what sort of services are available. To this end, two key initiatives have been proposed to address this gap:

- **Developing a National Mental Health Helpline:** The current Mental Health Helpline provides a resource largely for discharged patients from the main tertiary psychiatric hospital in Singapore. To extend the reach, the helpline will expand to become the National Mental Health Helpline to serve all. It serves as a first stop resource to provide information and education, and service navigation to both social providers and clients. It also provides crisis management.
- **A Mental Health Information Portal** will be developed to raise awareness on mental health issues to the public, clients and providers. Specifically, it will provide appropriate information to reduce the uncertainty of dealing with mental health issues and as a one stop for all national community resources and services, such as, support groups, job matching, etc.

While there is a clear need to strengthen Community Mental Health services in Singapore, the government recognises that mental health is often only a part of the array of issues which an individual faces. Hence, to be successful, mental health services need to be **integrated** with other health and social care services—i.e. weaving mental health services into other care services (e.g. primary care), including social services—so as to deliver a holistic approach to care that is person-centric. The evaluation of the outcomes of such an integrated system of care would need to encompass all elements including functional, personal and social dimensions. The road ahead to integrate mental health services is a long one. While the ultimate goal is to enable persons with mental illnesses to receive affordable and cost-effective clinical care in a coordinated and holistic manner, it remains to be seen if the approach outlined above will work or further fundamental changes will need to be made to the entire healthcare system.
